# Innovative appliance for colostomy patients: an interventional prospective pilot study

**DOI:** 10.1007/s10151-019-02059-x

**Published:** 2019-08-21

**Authors:** P.-A. Lehur, J.-B. Deguines, L. Montagliani, J.-P. Duffas, L. Bresler, F. Mauvais, K. Boudjema, E Chouillard, On behalf of the CC Study Group, M. F. Charenton, N. Daumard-Pasquier, C. Duval, I. Fernandes, M. T. Gatel, H. Sim, A. Souaré, A. Tourrou, E. Trividic

**Affiliations:** 1 Department of colorectal surgery, Clinique de chirurgie digestive et endocrinienne, Centre Hospitalier Universitaire, University hospital of Nantes, 1, place Alexis Ricordeau, 44,035 Nantes, France; 2 Centre Hospitalier, Service Chirurgie Digestive, Boulogne sur Mer, France; 3 Hôpital d’Instruction des Armées Begin, Service de Chirurgie Viscérale et Digestive, Saint Mandé, France; 4 CHRU Rangueil, Service de Chirurgie Générale et Digestive, Toulouse, France; 5 CHRU Brabois, Service de Chirurgie Digestive, Hépatobiliaire, Pancréatique, Endocrinienne et Cancérologique, Vandoeuvre Les Nancy, France; 6 CH Beauvais, Chirurgie viscérale et digestive, Beauvais, France; 7 CHU Pontchaillou, Service de Chirurgie Hépato-Biliaire et Digestive, Rennes, France; 8 Centre hospitalier, Service de Chirurgie Digestive Viscérale, Urologique et Plastique, Poissy/St Germain en Laye Hospital, Poissy, France

**Keywords:** Stoma care, Colostomy, Output control, Appliance, Body image, Colorectal surgery

## Abstract

**Background:**

The control of body waste emptying is a constant research topic in stoma care. The aim of this pilot study was to assess the efficacy and safety of an innovative colostomy appliance.

**Methods:**

An interventional prospective non-comparative pilot study was conducted in seven French centers. The study device is a new type of two-piece appliance including a base plate and a “capsule cap” (CC) composed of a capsule cover and a folded collecting bag. The device gently seals the stoma to provide stoma output control. When the bowel movement pressure increases the patient may control the deployment of the folded bag and collect stools. Patients with left-sided colostomy all using a flat appliance, were enrolled in a 2-week trial. Outcome measures were type of CC removal and peristomal fecal leaks while wearing the device.

**Results:**

Of 30 patients (females 66.7%), with left-sided colostomy (permanent 76.7%), 23 (76.7%) completed the 2-week trial. A total of 472 CC changes were analyzed. Efficacy: of 404 (85.5%) CC changes reported in diaries, 302 (74.8%) were linked with stool and/or gas. In 244 (60.3%) changes, the patient controlled stoma bag deployment and it occurred with bowel emptying 301 (74.5%) times. No leaks around the appliance were observed in 400 (85.3%) changes. Safety: no serious adverse event occurred. Peristomal skin was not modified during the trial.

**Conclusions:**

In the short term this new device has provided an increased control over bowel emptying at no risk in half of the trial population suggesting that an alternative approach to bag wearing is achievable.

## Introduction

It is estimated that as a result of disease, genetics or trauma, approximately 700,000 Europeans have had ostomy surgery to remove or divert diseased or damaged portions of their bowel or bladder [1]. Even though restorative colorectal surgery has been intensively developed, there are still a substantial number of patients that have to live the rest of their life with a permanent abdominal stoma [1]. A colostomy affects quality of life (QoL) of patients and families [2]. It implies significant changes physically and socially. Patients have to adjust to managing ostomy self-care and appliances and to deal with their new body image. Because of anxiety related to accidents and issues with stool leakage, odors and gas, many of them limit activities as they are worried about embarrassing situations in public [3]. They commonly feel their body out of control and report that pouch wearing has negative psychological impact [2, 3].

Today, ostomy appliances available are of one-piece or two-piece types. They make it possible to collect bowel effluents in a bag, managing gas and noise as far as possible and maintaining a healthy peristomal skin. Nevertheless, no alternative to a traditional external bag is currently unavailable, except colonic irrigation. Surveys have identified the absence of control over bowel effluent discharge as a major concern for patients [4]. Indeed, uncontrolled evacuation, noise, fear of odors and embarrassment with soiling affect negatively every aspects of daily life [5]. The ostomates still look for further improvement and a better adaptation of appliances to their lifestyle.

On-going research aims to further improve ostomy appliances and develop innovative technical means to regain control over body waste discharge while avoiding problems associated with pouch wearing. After several years of research, a new device (AOS-C2001-B) elaborated on an innovative concept of a stoma covering cap has been developed, tested and European Conformity (CE) marked. It is indicated for left colostomates having stool consistency ranking from hard to soft. The most essential part of this device is a “capsule cap” (CC) which contains a folded bag allowing collection of formed stool when deployed, either deliberately by the patient or spontaneously i.e. triggered by pressure within the bowel. This CC evolved through several design configurations studied in bench tests and clinical trial (ClinicalTrials.gov ID: NCT02602236) permitting improvement of its functionality that led to the present device. Wearing it could have a positive impact on the patient’s well-being, including body image and comfort. As part of a research and development program, a prospective pilot study was designed to assess device efficacy and safety when used by left colostomates (ClinicalTrials.gov ID: NCT03108105). As the device aims to allow control over bowel emptying, preferred outcome was assessed according to the mode of CC removal (manual, i.e. deliberately, or spontaneous) and the absence of fecal leaks with the stoma appliance. “Manual removal of the CC linked to a pressure feeling” and “no leaks” was the preferred outcome.

## Materials and methods

**Fig. 1:**
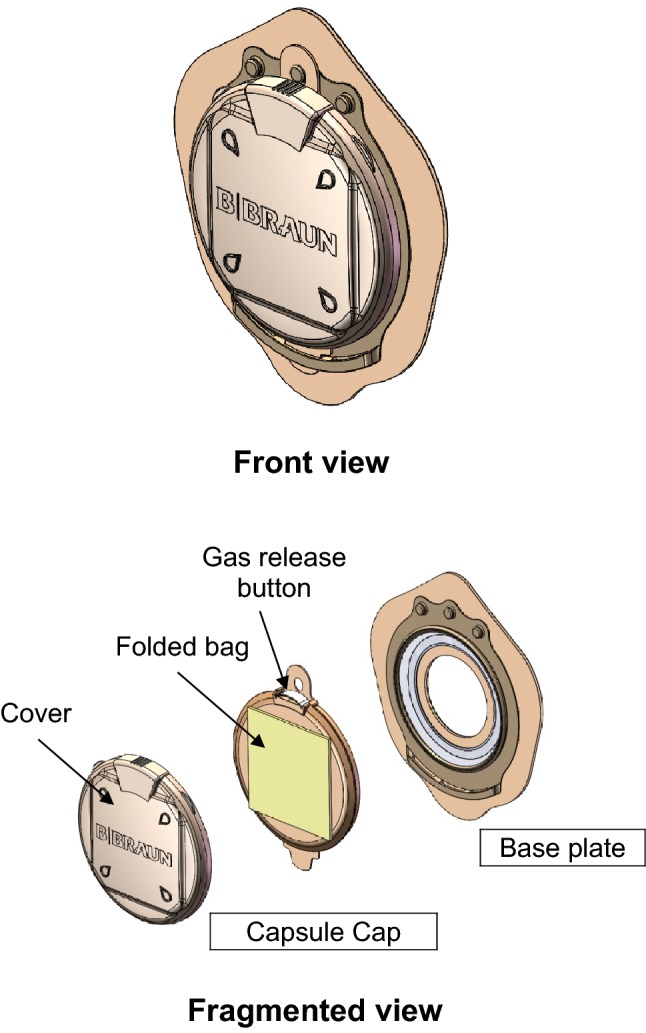
The investigational appliance AOS-C2001-B. Front and fragmented views. The capsule cap is composed of a capsule cover, a closed collecting multilayer-film folded pouch, a passive integrated filter for continuous release of flatus with a gas-release button to evacuate gas manually if necessary, a preliminary seal to hermetically close the stoma, and a coupling system with a guiding tab insuring a good positioning and a safe link of the capsule cap to a flat base plate (Flexima^®^ 3S or Flexima^®^ 3S/R Ø 55 or Ø 65 mm according to the stoma size)

The AOS-C2001-B device is a new concept: a two-piece appliance with an encapsulated bag. The CC is fixed on a Flexima^®^ 3S (B. Braun Medical) base plate (Fig. 1). It is delivered as a folded collecting bag enclosed within a capsule closed by a flexible cover containing a gas-release button and a filter. When intestinal pressure increases due to bowel movements, the cover bulges out and stiffens, indicating a need for pressure relief. Upon such need, the patient may either release gas or voluntarily remove the cover and thus deploy the bag. Then the stools expelled through the stoma are collected into the bag. When bowel emptying is completed, the bag can be disconnected from the base plate and discarded while a new CC is fixed on the same base plate.

### Study design

Participation to the trial lasted 2 weeks for each patient. Patients began to use the investigational device following full instruction by an enterostomal therapist (ET) at inclusion visit (V1). They were provided with devices (CC and Flexima^®^ 3S base plates) for a use of at least one CC per day. Standard Flexima^®^ 3S base plates were used at V1 by all patients. At the first base plate replacement (1–3 days after V1) patients could switch to a base plate variant “Flexima^®^ 3S/R” (“R” for “reinforced”) in case of detachment of the standard base plate and/or occurrence of sudden and massive leakage. During the study period (12 ± 2 days) the participants continued using either the standard or the reinforced base plate, after discussion with and following the advice of the ET. Participation to the trial ended at day 14 ± 3 (V2). Diaries completed by the patient at each CC change and questionnaires were collected by ETs.

### Patients

Adult (> 18 years old) patients with a left colostomy created more than 1 month previously, having formed stool and using flat ostomy appliances were eligible for inclusion. Patients had further to be able to apply and remove their appliance themselves, to understand the study procedures and to fill out diaries and questionnaires. Patients experiencing repeated leakage with their usual appliance, suffering from peristomal skin disorders, receiving chemotherapy, radiation therapy or steroids during the month preceding the study, or who had chronically liquid stools, were excluded.

### Endpoints

#### Efficacy

The primary preferred outcome of efficacy consisted in a combination of two endpoints to be recorded by the patients at each CC change on a diary. Namely, there were four possible types of CC change: (1) “manual CC removal, linked to pressure feeling”; (2) “manual CC removal, linked to another reason than pressure feeling”; (3) “spontaneous CC removal, linked to stools or gas”, (4) “spontaneous CC removal, not linked to stools or gas”. The second endpoint was the occurrence of leakage under the base plate, between the base plate and the CC or through the gas-release button. At each CC change, the patients recorded on the same diary the level of leakage they experienced during wear time as (1) “no leakage”; (2) “leakage not soiling clothes”; (3) “leakage soiling clothes”, (4) “sudden and massive leakage”—with the latter all three being accounted for as “leakage”. The preferred overall outcome to get full satisfaction was the combination of (1) “manual CC removal due to pressure feeling” and (2) “no leakage”.

#### Safety

Safety was evaluated by monitoring adverse events (AEs) and the condition of the peristomal skin at V1 and V2 using the validated Ostomy Skin Tool (DET score, 0–2 points) [6, 7].

#### Quality of life and satisfaction assessment

During V1 and V2 patient QoL was evaluated using the Stoma QoL questionnaire [8, 9]. Moreover, at the end of the study, patients were asked to evaluate the device performance in terms of application, discretion of the appliance, stoma-related noise during wear time, feeling of security and overall satisfaction including general impression before use, comfort of the appliance, and general impression about body image during use. Overall satisfaction with different parameters was recorded in the patient’s stoma diary at V2 using Likert 5-grade scale score (from “very good” to “very poor”). Since the CC device is a very new concept, a comparison with the current appliance was not relevant.

### Statistical analysis

Since this was a pilot study, the sample size was arbitrarily set at 30 patients and recruitment based on inclusion opportunities during the study period. Percentages were computed with 95% confidence interval (CI). The level of significance for all comparisons was set at 0.05. Missing data were not replaced and treated as such. All analyses were conducted using SAS software 9.2.

### Ethics

The study was conducted in accordance with the Declaration of Helsinki. It received Ethics Committee approval from the “Comité de Protection des Personnes” Tours Ouest 1, France, and Competent Health Authority authorization. Because the study was interventional research, all patients were duly informed by the investigator at each center and gave written consent to participate in the study.

## Results

### Baseline characteristics

Thirty patients (66.7% females) aged 63.7 ± 10.9 years having a left-sided colostomy created 3 years ago on average (2 months—10 years) mainly for colorectal cancer were included from May to October 2017 in 7 French colorectal centers (Table 1). Twenty-three (76.7%) and 7 (23.3%) patients respectively had a definitive or a temporary stoma. All patients wore flat appliances without repeated leaks with their current product. At inclusion, 23 and 6 patients used one-piece and two-piece appliances respectively, one using both alternatively. Five patients performed colonic irrigation on a regular basis. Mean follow-up time was 15.4 ± 5.2 days. All 30 patients were analyzed in the full analysis set population for efficacy parameters. Regarding peristomal skin condition with their usual appliance, no patient had discolored or eroded areas; 2 had moderate tissue overgrowth of less than 25% of peristomal area. The mean total DET score at inclusion was 0.13 ± 0.51.

**Table 1:** Demographics and stoma characteristics

	Total (*N* = 30)
Age (years)—mean ± SD [median]	63.7 ± 10.9 [63]
Female—*n* (%)	20 (66.7)
BMI (kg/m^2^)—mean ± SD [median]	26.65 ± 6.5 [24.63]
Colostomy history	
Time since stoma formation (months)—mean ± SD [median]	35.0 ± 34.8 [25.5]
Reason for formation—*n* (%)	
Colon or colorectal cancer	20 (66.7)
Fecal incontinence	1 (3.3)
Others^a^	9 (30)
Colostomy description	
Definitive stoma—*n* (%)	23 (76.7)
Left-sided—*n* (%)	30 (100)
Colonic irrigation	5 (16.6)
Stoma diameter (mm)—mean ± SD [median]	29.0 ± 5.3 [28]
Stoma height (mm)—mean ± SD [median]	5.5 ± 4.6 [4]
Effluent type—*n* (%)	
Solid	21 (70)
Other	9 (30)

*BMI* body mass index, *SD* standard deviation

^a^Perforation, diverticulitis, fistula

### Use modalities of the investigational device

In total 472 CC changes were performed. Mean CC wear time measured at each change was 7.53 h with bag folded [median = 5.50] and 8.98 h with bag deployed [median = 4.10].

### Efficacy

**Fig. 2:**
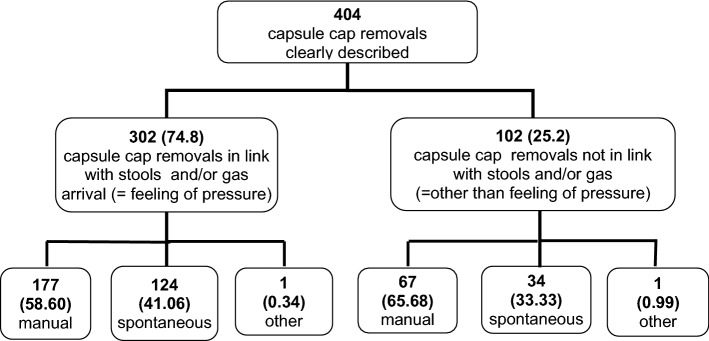
Modes of capsule cap removal

Of the 472 CC changes, 404 (85.5%) were clearly reported (Fig. 2): 302 (74.8%) were linked to a feeling of pressure due to output of stool and gas, whereas 102 (25.2%) were not. In 244 (60.3%) instances the patient removed the device manually. In these occasions he/she deliberately deployed the stoma bag and got control of the output. Overall, in 400 (85.3%) CC changes, the patient reported no leakage at all whereas in 69 changes there had been a leakage (1) without soiling clothes (*n* = 43 cases), (2) with soiling clothes (*n* = 20) and (3) with massive and sudden leakage (n = 6). The preferred outcome combining “spontaneous and manual CC removal due to pressure feeling or in link with bowel emptying” and “no leakage” was reached in 136 (28.9%) CC removals.

### Safety

There was no serious AE. Fourteen AEs of mild to moderate severity occurred in 9 (30.0%) patients. In 3, abdominal pain, diarrhea or dyschezia were considered as “possibly” to “certainly” related to the investigational device. These AEs resolved spontaneously and did not require discontinuation of the study. In 4 cases (13.3%), skin and subcutaneous tissue reaction were reported leading to premature study discontinuation. Two urinary tract infections were recorded.

There was no significant difference (*p* = 0.375) between total DET scores (mean ± SD), respectively, measured at V1 (0.13 ± 0.51) and V2 (0.43 ± 1.33).

### Device acceptability and QoL

Whether or not the device was appreciated by patients was also explored (Table 2): 90% had “good” to “very good” impression of the device before use; 76.7% found the CC application “easy” to “very easy”; 79.3% rated stoma-related noise during the wear time as “not very noisy” to “not noisy at all”; 66.6% found the appliance “comfortable” or “very comfortable” and 70% had a “good” to “very good security feeling” with the device. Then, 60% had “good” to “very good” impression of their body image during the use.

**Table 2:** General impression during the use of the investigational device (FAS/*n* patients = 30)

Overall security feeling *n* (%)	Very good	5 (16.7)
	Good	16 (53.3)
	Bad	8 (26.7)
	Very bad	1 (3.3)
Overall satisfaction *n* (%)	Very satisfactory	3(10)
	Satisfactory	12 (40)
	Not very satisfactory	11 (36.7)
	Not satisfactory at all	4 (13.3.7)
General impression of body image *n* (%)	Very good	4 (13.3)
	Good	14 (46.7)
	Bad	9 (30)
	Very bad	3 (10)

There was no statistically significant change in the global Stoma QoL score between V1 and V2 (59.18 ± 11.24 (V1)—60.95 ± 10.11 (V2), *p* = 0.907). Finally, 50% of patients were overall “satisfied” to “very satisfied” with the appliance and 43% were willing to use it in the future (Table 2).

## Discussion

This clinical study was set up to measure the efficacy and safety of an innovative device designed so that patients could regain an almost continent state by controlling the bowel output. The targeted sample size of 30 colostomates was reached allowing assessment of 472 CC removals. In almost 75% of cases, patients were able to control gas by pressing the release button and stool emission by manually removing the CC to deploy the bag. Over the short course of the study, the preferred outcome was achieved in nearly 30% of CC deployment, patients considering positively the gain of a related–stoma output control occurring without leakage. These results are more than encouraging when we know that the use of a new device involves a learning curve and adaptation in a patient’s daily routine. Moreover, it is well recognized that leakage is the criterion that has the highest impact on QoL [4]. In our study, more than 85% of cases reported no leakage during the device wearing period. However, as no comparable device exists on the market, it is difficult to compare to the level of leakage reported in clinical trials conducted in colostomates with the two-piece system. Nevertheless, in different trials, the leakage rate occurring under the base plate was collected: one study conducted on colostomates reported the absence of leakage in 70% of bag changes with one-piece flat standard appliance (ClinicalTrials.gov ID: NCT01243294). Based on these data, our results suggest a similar efficacy of the new device in that respect.

Due to a number of anatomical and physiological reasons, it has been so far impossible for colostomates to regain fecal continence. For this population redefining bowel continence was suggested as “the ability to gain some level of control and predictability of fecal elimination” [10]. Colostomates have difficulty accepting incontinence and moving on with life as they often feel stigmatized by family, society or employers. Most of them would very much appreciate being able to regain almost complete control over discharge of stool and gas [2, 3, 10]. Therefore, the investigational appliance device was optimally designed to collect *formed* effluent allowing colostomates to control their bowel movements and to trigger stool evacuation. Its use is excluded for patients with ileostomies, wet or diverting colostomies, or in case of important abdominal deformity linked to a parastomal hernia.

One of the best examples of surgically designed “bowel continence restoration” when constructing a stoma is the “Koch pouch continent ileostomy” allowing for a regular emptying of bowel effluent for ileostomates [11]. Non-surgical strategies for stoma continence have been widely used based on colostomy irrigation or plug or a combination of both developed by some companies. Instead of collecting effluents in a bag, they offer a possibility for colostomates to trigger complete bowel evacuation once every 24–48 h and then to wear a stoma plug rather than a pouch in between irrigations. This procedure is considered very time-consuming, however, and not completely satisfying for many stoma patients [10]. A pouchless colostomy continence device (Vitala^®^) had been developed and marketed (ConvaTec, Skillman NJ, USA). This device that provided temporary continence but had met with limited success among the targeted patients, is no longer available [12–15].

In the literature, it is well described that stoma appliance influences the patients’ daily life and body image [16, 17]. Therefore, data reported by the patient’s own rating of the study device are a good indicator of performance and reflect physical well-being. In this study, it was shown that 70% of patients rated the overall feeling of security as “good” or “very good”, 67% when evaluating how comfortable the device was to wear answered “good” or “very good” and 60% had a “good” to “very good” general impression of their body image during the use of the device. The alteration of the body image after stoma creation implies a change in awareness of both appearance and function of the individual [18]. With the current findings, we can postulate that AOS-C2001-B is a real opportunity for colostomates to improve self-esteem and well-being.

Adoption of the new device has been challenging for the study patients as a majority (77%) were using a one-piece appliance prior entering the study, which by definition is more discreet than a two-piece appliance. Consequently the device was considered discrete (not bulky) by only 40%, even if 79% judged that the device is soundless and prevents stoma-related noises. Some problems associated with pouch use such flatus, odor, and noise can often become stressful and embarrassing for the patient [15]. Present results show that the device could influence patients’ daily life and allow them to recover a better social life including leisure activities.

As regards safety, no serious AEs occurred and only a few AEs were considered “possibly” to “certainly” related to the investigational device. These skin and subcutaneous complications are well known and occurred less often than those reported in other clinical trials performed with a two-piece appliance (ClinicalTrials.gov, ID: NCT02362360). In this randomized, cross-over study adverse events for skin and subcutaneous tissue occurred in 17/49 (34.7%) and 9/51 (17.7%) cases in the comparator group and the test group, respectively. Therefore, skin around the stoma was not threatened by the device as shown by an unmodified DET score in the course of the study. The small number of leakages reported during the study period show that the peristomal skin was well protected.

Finally, this study suggests that the patient wearing this new device could control the output discharge and regain such of bowel continence as defined by Robert [10]. This is obtained without any surgical modification in raising the colostomy if the current recommendations are followed [19]. It offers a good alternative to bag wearing with a real impact on the patient’s QoL including a better self-perception of his/her body image.

Several limitations may have influenced the final study results. Firstly, the number of patients in whom the CC device has been tested is small. It was not possible for instance to compare for “early users” to those with an “old” stoma with regard to the impact of the new device. Thus the study has to be considered as preliminary to a larger trial. However, more than 400 CC were used by the 30 patients over the 2-week period allowing for a good evaluation of the device itself. Secondly, study period was short. Outcome and acceptance over longer periods of time must be investigated. A learning curve effect could not be evaluated in such a short time span, but considering similar studies, it is expectable that results may improve with time as the patient gets more familiar with the device and its interaction with his/her stoma [14]. The impact of this new device on patients’ QoL remains also to be assessed over a longer time span. Nevertheless, the data collected are particularly encouraging considering the breakthrough technology of the AOS-C2001-B in the absence of any comparator on the market.

## Conclusions

In this pilot study, the efficacy and safety of a novel output control device for left colostomates has been confirmed in the short term, with a significant number of patients ready to use it for longer periods of time. Such an alternative to bag wearing could improve bowel control, stoma acceptance and patients’ QoL. The present report paves the way for further studies exploring the benefits of voluntary control of stool evacuation in colostomates.

## Abbreviations

CC: Capsule cap
AEs: Adverse events
NA: Not available
QoL: Quality of life
DET score: Discoloration, erosion, tissue overgrowth score
ET: Enterostomal therapist
FAS: Full analysis set
